# Obinutuzumab and Chemotherapy for Front-Line Treatment of Follicular Lymphoma Grade 3B and Composite Follicular Lymphoma: A Study of the Croatian Cooperative Group for Hematologic Diseases (KroHem)

**DOI:** 10.3390/ph19101640

**Published:** 2026-10-18

**Authors:** Davor Galusic, Neno Zivkovic, Sandra Basic-Kinda, Barbara Dreta, Marija Ivic, Dino Dujmovic, Martina Moric-Peric, Hrvoje Holik, Neven Franjic, Jasminka Sincic-Petricevic, Ida Hude-Dragicevic, Igor Aurer

**Affiliations:** 1 University Hospital of Split, Soltanska 1, 21000 Split, Croatia; 2 School of Medicine, University of Split, 21000 Split, Croatia; 3 University Hospital Centre Zagreb, 10000 Zagreb, Croatiaigor.aurer@mef.hr (I.A.); 4 University Hospital Dubrava, 10000 Zagreb, Croatia; 5 General Hospital Zadar, 23000 Zadar, Croatia; 6 General Hospital Dr. Josip Bencevic, 35000 Slavonski Brod, Croatia; 7 University Hospital Centre Rijeka, 51000 Rijeka, Croatia; 8 School of Medicine, University of Rijeka, 51000 Rijeka, Croatia; 9 University Hospital Centre Osijek, 31000 Osijek, Croatia; 10 School of Medicine, University of Zagreb, 10000 Zagreb, Croatia

**Keywords:** obinutuzumab, follicular lymphoma grade 3b, composite lymphoma, chemotherapy, survival, prognosis

## Abstract

**Background:** Data on outcomes of follicular lymphoma grade 3B (FL gr.3B) and composite lymphoma (FL/large B-cell lymphoma (B-LCL)) are scarce and the effect of obinutuzumab is unknown. **Patients and methods:** We analyzed outcomes of patients with FL gr.3B & FL/B-LCL receiving front-line treatment with obinutuzumab-chemotherapy combinations and compared them to previously published cohorts with FL gr.1–3A and diffuse large B-cell lymphoma (DLBCL). **Results:** Twenty-six patients were included; nine had FL gr.3B and 17 had FL/B-LCL. Response rate was 92%, and 60% achieved complete remission. OS and PFS rates at 3 years were 76% and 67%, respectively. Outcomes of patients with FL gr.3B and FL/B-LCL were similar. PFS and OS rates at 3 years were both 100% in patients with FLIPI 0–2 vs. 42% and 61% in those with FLIPI 3–4 (*p* = 0.005 and *p* = 0.031). PFS and OS rates at 3 years were both 100% in patients with IPI 0–2 vs. 29% and 55%, respectively, in those with IPI 3–5 (*p* = 0.004 and *p* = 0.014). Low-risk patients with FL gr.3B & FL/B-LCL, FL gr.1–3A and DLBCL all had 3-year OS and PFS rates above 80%. High-risk patients with FL gr.3B & FL/B-LCL had outcomes inferior to those with FL gr. 1–3A and similar to those with DLBCL. **Conclusions:** Our results suggest that front-line therapy with obinutuzumab-chemotherapy combinations has excellent outcomes in patients with low-risk FL gr.3B & FL/B-LCL, that both FLIPI and IPI are of prognostic value and that the clinical course of FL gr.3B & FL/B-LCL is more similar to DLBCL than FL gr.1–3A.

## 1. Introduction

Follicular lymphoma grade 3B (FL gr.3B) is a rare subtype of FL characterized by a more aggressive clinical course, genetically resembling diffuse large B-cell lymphoma (DLBCL) more than indolent FL (gr.1–3A) [1,2,3,4,5,6,7,8]. It is usually excluded from clinical trials of FL [9,10,11], but sometimes included in those of DLBCL [12]. In the fifth edition of the World Health Organization classification, the name of this entity was changed to follicular large B-cell lymphoma, while the International Consensus Classification kept the standard nomenclature of FL [13,14]. Composite FLs are lymphomas with both FL and DLBCL components coexisting synchronously in biopsy samples (FL/B-LCL) [1,15]. There is no consensus regarding optimal treatment of these entities. Published data are scarce and generally retrospective. Guidelines recommend using more aggressive chemotherapy regimens, designed for DLBCL, with the addition of the anti-CD20 antibody rituximab (R) [16,17]. However, some published reports of patients treated with rituximab-based therapies suggest that the clinical outcomes are more similar to those of indolent FL than DLBCL [18,19]. The optimal choice of anti-CD20 antibody for FL gr.3B & FL/B-LCL also remains unresolved. The large multicenter randomized study GALLIUM demonstrated that, in patients with FL gr.1–3A receiving front-line treatment, combinations of obinutuzumab (G) with chemotherapy improve response rates (RRs) and progression-free survival (PFS) in comparison to combinations of R with chemotherapy [11]. In contrast, GOYA, a similar study performed in previously untreated DLBCL patients, showed no advantage of G over R [20]. However, detailed analysis suggested that a subgroup of DLBCLs with molecular characteristics very similar to FL had a better RR if treated with obinutuzumab, but due to the nature of the subgroup analysis and difficulties in defining these patients in routine clinical practice, this finding was not further pursued [21]. There are no data on the effect of obinutuzumab-based regimens in FL gr.3B or FL/B-LCL.

In Croatia, obinutuzumab is reimbursed for first-line therapy of all FL types. Some centers use it also for treatment of FL gr.3B and FL/B-LCL. In order to describe the outcomes of these patients and elucidate the possible role of obinutuzumab in the therapy of FL gr. 3B and FL/B-LCL, KroHem, the Croatian Cooperative Group for Hematologic Diseases, collected data on outcomes of all such patients treated between 2019, when G became reimbursable, and 2023.

## 2. Results

We identified 26 patients fulfilling entry criteria, 15 male (58%) and 11 female (42%). Patient characteristics are presented in Table 1. Median age was 62 years, ranging from 22 to 85. Nine patients (35%) had FL gr.3B and 17 (65%) had FL/B-LCL. Twelve patients were hospitalized for the first administration of G, and 14 were not. The median number of both G and chemotherapy cycles was 6, ranging from 1 to 6. Five patients (19%) stopped treatment prematurely, four due to toxicity (three infections, one hyperglycemia) and one due to progressive disease. The overall RR was 92%; 15 patients (60%) achieved complete remission, one had progressive disease, and one died of infection prior to response evaluation. Most common side effects were hematologic and infectious. Anemia gr. 3–4 occurred in 6 patients (23%), thrombocytopenia gr. 3–4 in 6 (23%), and neutropenia grade 3–4 in 11 (42%). Additionally, 35% of patients (*n* = 9) experienced grade 3–5 infections, primarily driven by Severe Acute Respiratory Syndrome Coronavirus 2 (SARS-CoV-2). A more detailed list of adverse events was not provided because safety reporting during the COVID-19 pandemic was limited. After induction, 20 patients continued with G maintenance.

**Figure 1:**
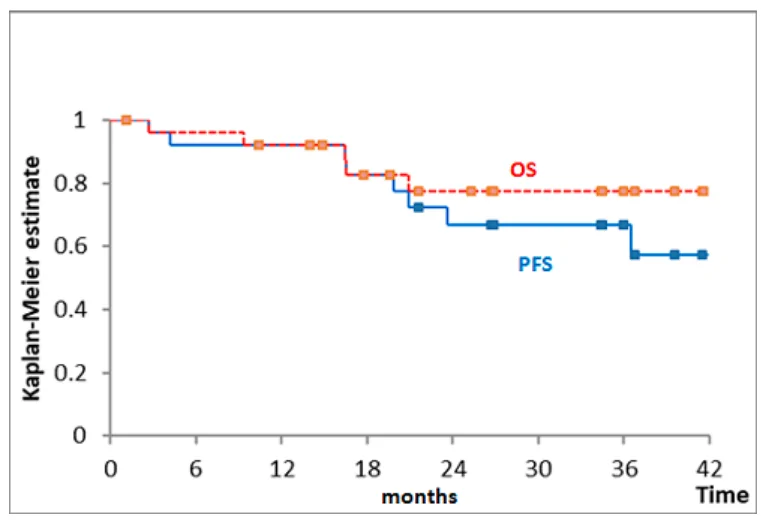
OS and PFS of the whole cohort.

**Figure 2:**
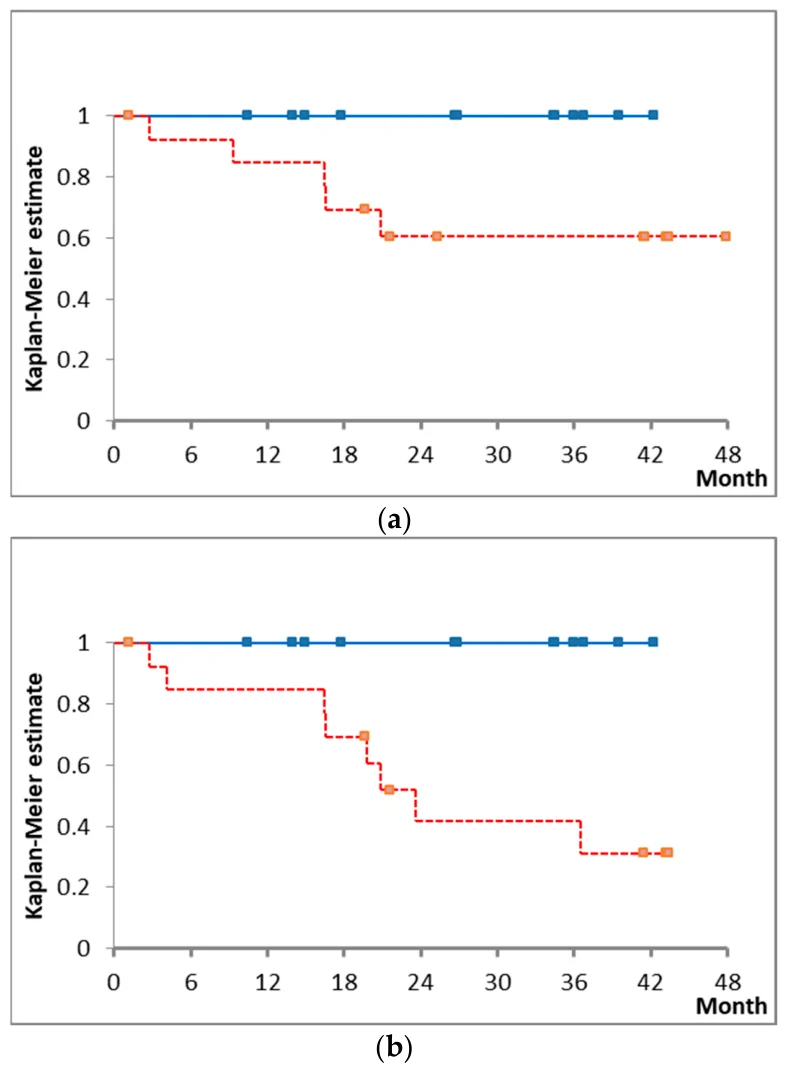
(**a**) OS according to FLIPI (*p* = 0.031). Blue line: FLIPI 0–2, red line: FLIPI 3–4. (**b**) PFS according to FLIPI (*p* = 0.005). Blue line: FLIPI 0–2, red line: FLIPI 3–4.

**Figure 3:**
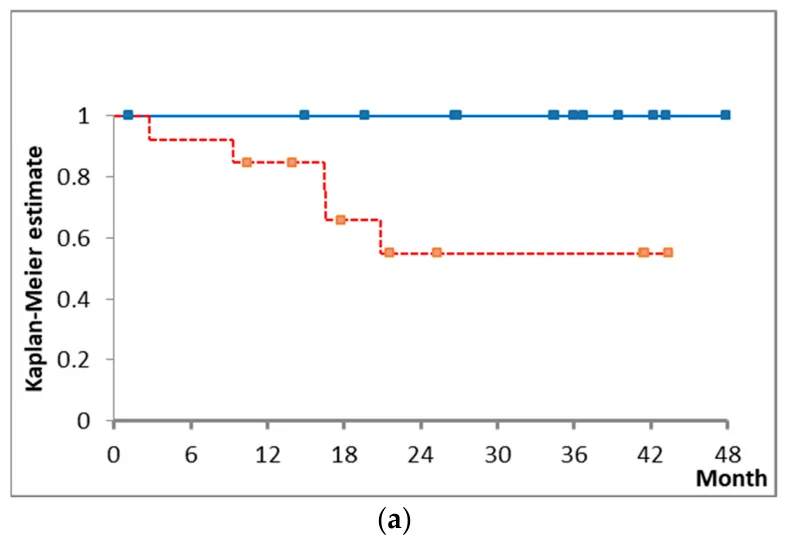

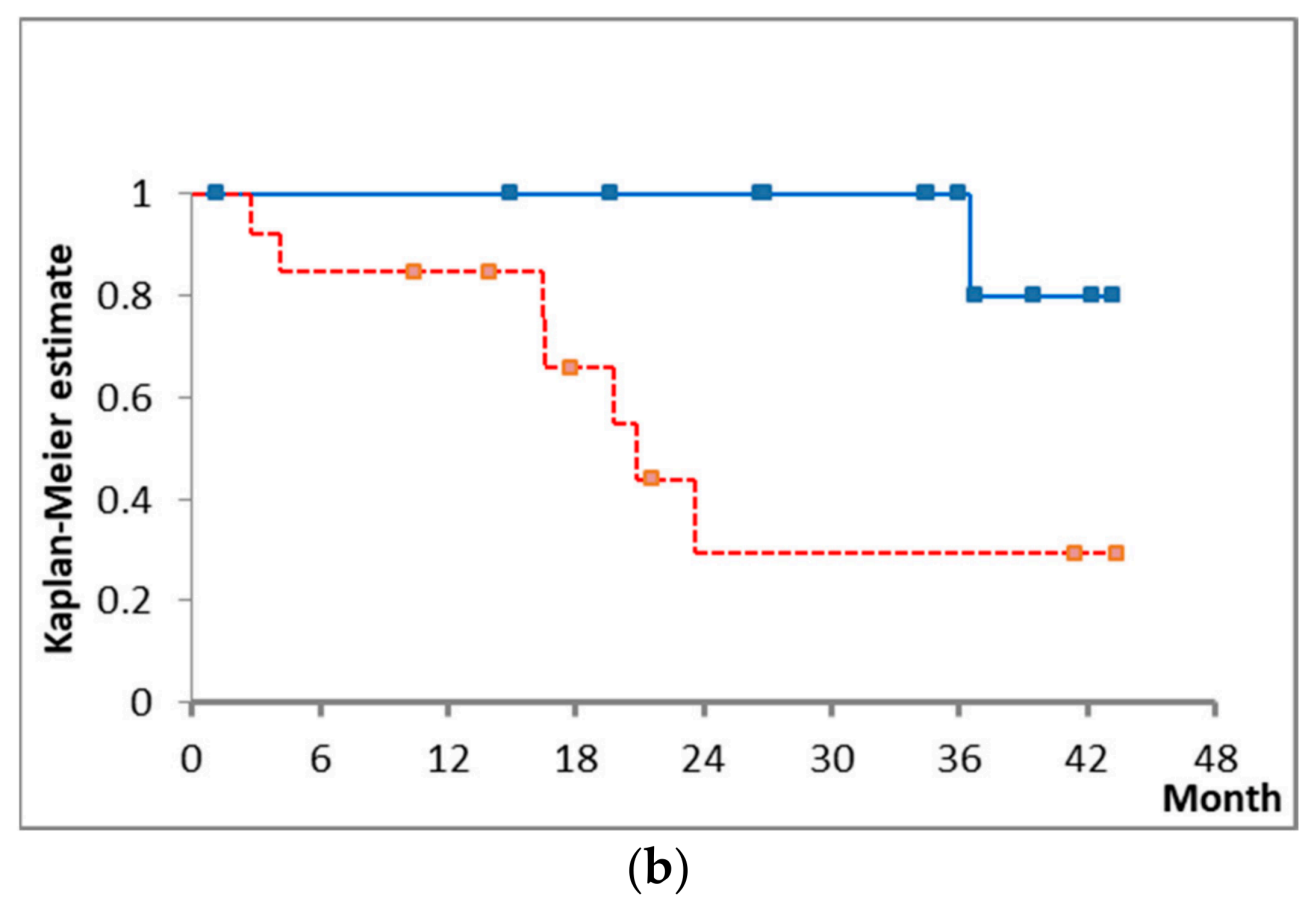
(**a**) OS according to IPI (*p* = 0.014). Blue line: IPI 0–2, red line: IPI 3–5. (**b**) PFS according to IPI (*p* = 0.004). Blue line: IPI 0–2, red line: IPI 3–5.

After a median follow-up of 34 months with the survivors, 4 patients progressed and 5 died. OS and PFS rates at 3 years were 76% and 67% (95% confidence interval (CI) 60–95% and 46–87%), respectively (Figure 1). Outcomes of patients with FL gr.3B and FL/B-LCL were similar. Age and sex did not influence outcomes. Patients with lower FLIPI and IPI had significantly better outcomes. PFS and OS rates at 3 years were both 100% in patients with FLIPI 0–2 vs. 42% and 61% (95% CI 13–70% and 33–88%), respectively, in those with FLIPI 3–5 (*p* = 0.005 (HR 7.49) and *p* = 0.031 (HR 6.88), respectively) (Figure 2). PFS and OS rates at 3 years were both 100% in patients with IPI 0–2 vs. 29% and 55% (95% CI 0–60% and 25–85%), respectively, in those with IPI 3–5 (*p* = 0.004 (HR 10.97) and *p* = 0.014 (HR 9.31), respectively) (Figure 3).

Details of the comparator groups with indolent FL and DLBCL were published previously [22,23]. From the former group, the cohort of indolent FL patients treated with G-CHOP or G-CVP, but not G-bendamustine, was used for comparison. From the latter group, the cohort consisting of all DLBCL patients treated with R-CHOP-like regimens was used for comparison. Low-risk patients in all three groups had excellent outcomes with 3-year PFS and OS rates above 80% (Table 2).

**Figure 4:**
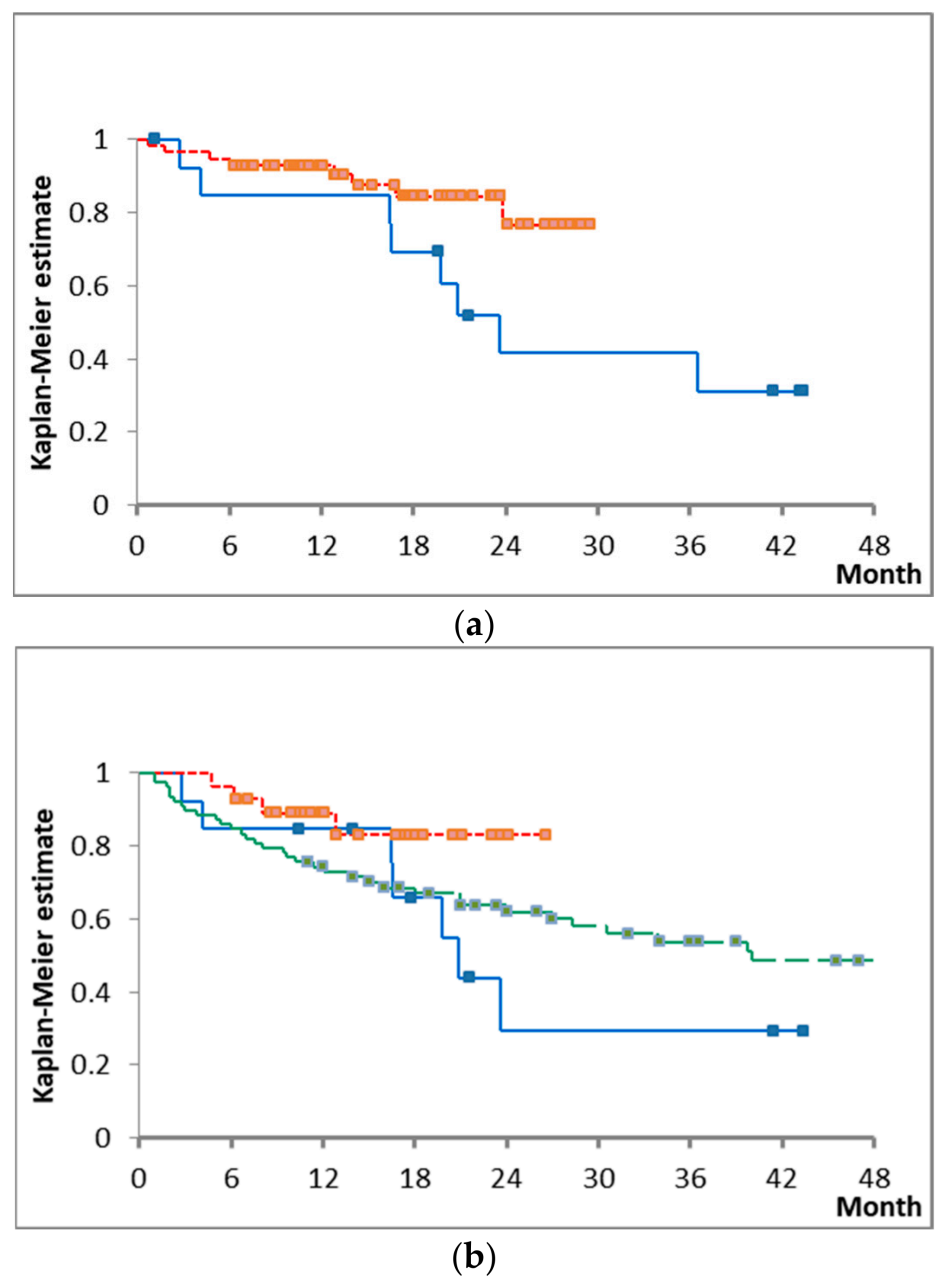
(**a**) PFS of FL grade 1–3A (red line), and FL gr.3B & FL/B-LCL (blue line) with FLIPI 3–4 (*p* = 0.034). (**b**) PFS of FL grade 1–3A (red line), FL gr.3B & FL/B-LCL (blue line), and DLBCL (green line) with IPI 3–5 (*p* = 0.075).

In contrast, those with FL gr.3B & FL/B-LCL and FLIPI 3–5 had a significantly inferior PFS in comparison to the similar group of patients with FL gr.1–3A (*p* = 0.034) (Figure 4a). PFS of patients with FL gr.3B & FL/B-LCL and IPI 3–5 was similar to that of DLBCL patients with IPI 3–5 (*p* = 0.416). There was a trend towards inferior PFS of FL gr.3B & FL/B-LCL patients with IPI 3–5 in comparison to that of FL gr. 1–3A and IPI 3–5 (*p* = 0.075) (Figure 4b). Differences in OS of high-risk patients from the three groups were less pronounced and failed to reach statistical significance. Details regarding indolent and aggressive lymphoma comparator groups were provided in Supplemental Tables S1 and S2.

## 3. Discussion

Our results suggest that combinations of G and chemotherapy, followed by G maintenance, produce excellent outcomes in patients with low-risk FL gr.3B & FL/B-LCL. The outcome of high-risk patients is far less favorable.

There are very few published reports on the outcomes of FL gr.3B or FL/B-LCL in the anti-CD20 monoclonal antibody era. We were unable to find any specifically describing the effect of G-chemo combinations in these patients. The most comprehensive report on front-line therapy of FL gr.3B & FL/B-LCL is a study from Australia, Canada and the United Kingdom, which included patients who received either G or R in combination with CHOP-like chemotherapy, but it did not report outcomes separately according to the anti-CD20 monoclonal antibody type [18]. The study included 157 patients with FL gr.3B or FL/B-LCL who started treatment between 2002 and 2019 and compared their outcomes to those of contemporary diagnosed FL gr.3A patients from all participating centers and Australian DLBCL patients. They found that FL gr.3A and FL gr.3B & FL/B-LCL patients had similar outcomes, superior to those of DLBCL. However, the FL gr.3B & FL/B-LCL group had the lowest FLIPI and IPI scores and, despite the fact that the difference in OS between different FLIPI-risk groups did not reach statistical significance (*p* = 0.16), there was a clear separation of curves between those with FLIPI 0–2 and FLIPI 3–5, much like in our series. Several other retrospective studies compared FL gr.3B & FL/B-LCL to FL gr.3A and had conflicting results. One, including 17 FL gr.3B patients, showed that they have outcomes similar to DLBCL and worse than FL gr.3A patients treated with R-CHOP-like regimens [17]. In this series, both IPI and FLIPI scores were of prognostic value, similar to our results. Two other studies yielded opposite results. In one, including 22 FL gr.3B patients, their outcome was the same as that of FL gr3A, despite the fact that the former had more high-risk disease (IPI 3–5) [24]. However, it should be noted that FL gr.3B patients more frequently received anthracycline-based chemotherapy. In the other study, 21 FL gr.3B & FL/B-LCL patients had outcomes similar to FL gr.3A patients and FLIPI was not of prognostic significance in either of the groups [19]. The reasons for these discrepancies remain unclear. Possible explanations include differences in pathological grading assignment or induction and post-induction therapy. In comparison to cohorts from two major clinical trials of G-chemotherapy combinations (GALLIUM and GOYA), our results are more similar to those of the GOYA study, additionally supporting the notion that FL gr.3B & FL/B-LCL share greater similarities with DLBCL than with FL gr.3A [11,20].

The main limitation of this study is the small sample size, which reduces statistical power and compromises the stability of survival estimates. The lack of central pathological review is also an important source of bias. Furthermore, in the absence of a rituximab-treated arm, our study cannot determine the real benefit of obinutuzumab over rituximab in this “borderline” setting. The comparisons with indolent and aggressive lymphoma cohorts, treated with very similar obinutuzumab- and rituximab-based therapy, were intended primarily to provide clinical context for this therapeutically challenging transitional entity, rather than statistically compare these uncontrolled heterogeneous cohorts. The chemotherapy regimens used by different centers were not uniformly consistent and were based on the clinical decision of the hematologist. Treatment choice, heterogeneity of cohorts and timing of these studies might seem confounding, but it reflects real-world clinical practice. Only a very small proportion of our patients with FL gr.3B did not receive G maintenance therapy; therefore, the independent impact of maintenance therapy on PFS and OS could not be evaluated.

## 4. Materials and Methods

Patients with FL gr.3B and FL/B-LCL, diagnosed at reference hematopathological centers, receiving front-line treatment with G in combination with any chemotherapy regimen, were included in this analysis. Data were collected retrospectively by chart review from hospital electronic databases in the period between January 2019 and December 2023. These included pretreatment patient and disease characteristics (age, sex, performance status, blood counts, lactate dehydrogenase (LDH) level, disease stage, number of involved nodal areas and extranodal localizations), treatment regimen, side effects, RR, PFS and overall survival (OS). Diagnostic procedures, treatment, response assessment and follow-up were performed according to national clinical practice guidelines [25]. Pretreatment staging was performed by PET-CT or CT scans and bone marrow biopsy. Response assessment after induction therapy was performed by PET-CT; bone marrow biopsies were repeated if they were initially positive. Imaging studies were not repeated during follow-up unless clinically indicated. Response to therapy was evaluated according to the Lugano and/or RECIL criteria [26,27]. OS was defined as the period from treatment start to death irrespective of cause. PFS was defined as the period from treatment start to treatment failure (including insufficient response and progression) or death. Descriptive statistics, available within the Microsoft Excel 2016^®^ program, were used to summarize baseline characteristics. Survival curves were plotted using the Kaplan–Meier method and their differences were compared with the log-rank test using a freely available statistical program [28]. Toxicity was graded retrospectively by chart review according to CTCAE v.5 criteria [29]. The study was approved by the Ethics Committee of the University Hospital Centre Split, number 2181-147/01/06/M.S.-22-02. Since this was a retrospective study of anonymized patient data, a waiver of the requirement for informed consent was obtained, and the identity of patients was never disclosed. The outcomes of the FL gr.3B & FL/B-LCL group were compared to those of contemporary patients with indolent FL treated with G-CHOP/CVP and DLBCL treated with R-chemotherapy combinations between 2011 and 2019. The characteristics and outcomes of these patients were described in detail previously [22,23].

## 5. Conclusions

It is impossible to draw firm conclusions on the value of G and anti-CD20 maintenance therapy in FL gr.3B & FL/B-LCL from this small study. Still, our results suggest that patients with low-risk disease (i.e., IPI 0–2 or FLIPI 0–2) receiving G-chemotherapy combinations followed by G maintenance have excellent outcomes. Additional studies are needed to define optimal therapeutic approaches for those with high-risk disease.

Our data suggest that FL gr.3B & FL/B-LCL is clinically more similar to DLBCL than to indolent FL. However, due to discrepant results of different studies, in order to elucidate the clinical importance of the distinction between FL gr.3A and 3B and define optimal therapeutic approaches for the latter group, larger, international efforts are needed. Due to the scarcity of the entity, prospective clinical studies are probably unrealistic. Establishing an international registry seems to be the best approach, but it would have to be coupled with a comprehensive pathological review of biopsy samples.

## Figures and Tables

**Table 1:** Patient characteristics.

N	26
Age (median/range)	62 y/22–85 y
Sex (M/F)	15/11
FL gr.3B/FL/B-LCL	9/17
FLIPI (0–2/3–5)	12/14
IPI (0–2/3–5)	13/13
Chemotherapy regimen G-CHOP G-DA-EPOCH G-CHOEP14 G-CEOP	16 8 1 1
Maintenance therapy	20

FL gr.3B—follicular lymphoma grade 3B, FL/B-LCL—composite follicular lymphoma FLIPI—The Follicular Lymphoma International Prognostic Index, IPI—The International Prognostic Index, G-CHOP—cyclophosphamide, doxorubicin, vincristine, prednisone and obinuzumab, G-DA-EPOCH—dose-adjusted etoposide, prednisone, vincristine, cyclophosphamide, doxorubicin, and obinutuzumab, G-CHOEP14—cyclophosphamide, doxorubicin, vincristine, prednisone, etoposide and obinutuzumab, G-CEOP—cyclophosphamide, vincristine, prednisone, etoposide and obinutuzumab.

**Table 2:** Comparison of outcomes of patients with FL gr.3B & FL/B-LCL, FL gr.1–3A and DLBCL.

	N	FLIPI 0–2		IPI 0–2		FLIPI 3–5		IPI 3–5	
Outcomes at 2 Years		PFS	OS	PFS	OS	PFS	OS	PFS	OS
FL gr.3B & FL/B-LCL	26	100%	100%	100%	100%	42%	61%	29%	55%
FL gr.1–3A	53	89%	94%	84%	94%	80%	96%	100%	100%
DLBCL	285			91%	91%			62%	70%
*p* values									
FL gr.3B & FL/B-LCL vs. FL gr.1–3A		0.303	0.453	0.177	0.219	0.034	0.133	0.075	0.118
FL gr.3B & FL/B-LCL vs. DLBCL				0.908	0.277			0.416	0.632
FL gr.1–3A vs. DLBCL				0.520	0.833			0.081	0.146

FL gr.3B—follicular lymphoma grade 3B, FL/B-LCL—composite follicular lymphoma, FL gr.1–3A—follicular lymphoma grade 1–3A, DLBCL—diffuse large B-cell lymphoma, FLIPI—The Follicular Lymphoma International Prognostic Index, IPI—The International Prognostic Index, PFS—progression-free survival, OS—overall survival.

## Data Availability

The original contributions presented in this study are included in the article/Supplementary Material. Further inquiries can be directed to the corresponding author.

## Supplementary Materials

The following supporting information can be downloaded at: https://www.mdpi.com/article/10.3390/ph19101640/s1, Table S1: patient characteristics of indolent lymphoma comparator group. Table S2: patient characteristics of aggressive lymphoma comparator group.
