# Supplementary material for: Obinutuzumab and Chemotherapy for Front-Line Treatment of Follicular Lymphoma Grade 3B and Composite Follicular Lymphoma: A Study of the Croatian Cooperative Group for Hematologic Diseases (KroHem)
Source: Pharmaceuticals (Basel). 2026 Oct 18;19(10):1640. doi: 10.3390/ph19101640 (PMC13708238; doi:10.3390/ph19101640)
Supplement: Supplementary file 1 [file pharmaceuticals-19-01640-s001.zip › Table S1 patient characteristics of indolent lymphoma comparator group.pdf]

N	53
Age (median / range)	63 y / 33-86 y
Sex (M / F)	20 / 33
FL gr.1 / gr.2 / gr.3A	13 / 15 / 25
FLIPI (0-2 / 3-5)	30 / 23
IPI (0-2 / 3-5)	41 / 12
Chemotherapy regimen	
G-CHOP	49
G-CVP	4

FL gr.1 – follicular lymphoma grade 1, FL gr.2 - follicular lymphoma grade 2, FL gr.3A - follicular lymphoma grade 3A, FLIPI - The Follicular Lymphoma International Prognostic Index, IPI – The International Prognostic Index, G-CHOP - cyclophosphamide, doxorubicin, vincristine, prednisone and obinuzumab, G-CVP - cyclophosphamide, vincristine, prednisone, and obinutuzumab
