# Supplementary material for: Obinutuzumab and Chemotherapy for Front-Line Treatment of Follicular Lymphoma Grade 3B and Composite Follicular Lymphoma: A Study of the Croatian Cooperative Group for Hematologic Diseases (KroHem)
Source: Pharmaceuticals (Basel). 2026 Oct 18;19(10):1640. doi: 10.3390/ph19101640 (PMC13708238; doi:10.3390/ph19101640)
Supplement: Supplementary file 1 [file pharmaceuticals-19-01640-s001.zip › Table S2 patient characteristics of aggressive lymphoma comparator group.pdf]

B - LCL	N = 285
Age (median / range)	59 y / 18 - 87 y
Sex (M / F)	145 / 140
IPI (0-2 / 3-5)	138 / 147
Chemotherapy regimen	
R-CHOP	137
DA-R-EPOCH	36
R-CHOEP	81
R-CEOP or R-miniCHOP	31

B - LCL – B-large cel lymphoma, IPI – The International Prognostic Index, R-CHOP - cyclophosphamide, doxorubicin, vincristine, prednisone and rituximab, DA-R-EPOCH – dose adjusted etoposide, cyclophosphamide, vincristine, doxorubicin, prednisone and rituximab, R-CHOEP - cyclophosphamide, doxorubicin, vincristine, etoposide, prednisone and rituximab, R-CEOP - cyclophosphamide, etoposide, vincristine, prednisone and rituximab.
